# Espaços de cura no mundo lusófono: sanatórios portugueses e o debate higienista

**DOI:** 10.1590/S0104-59702024000100045

**Published:** 2024-10-11

**Authors:** Cybelle Salvador Miranda, Renato da Gama-Rosa Costa

**Affiliations:** i Professora, Universidade Federal do Pará. Belém – PA – Brasil cybelle1974@hotmail.com; ii Pesquisador, Casa de Oswaldo Cruz/Fiocruz. Rio de Janeiro – RJ – Brasil gamarosacosta@gmail.com

Resultado de sua tese de doutoramento, agraciada em 2018 com o Prêmio Victor Sá de História Contemporânea, o livro de Avelãs Nunes, premiado com a “Lusitânia” da Academia Portuguesa em História em 2022, revela-nos um universo particular da história da saúde em Portugal, mas de forte conexão com outros países, incluindo com o Brasil, por conta da abrangência da tuberculose como um dos piores flagelos do século XX. Estudo francês, também resultado de uma tese de doutorado, *Architecture thérapeutique, histoire des sanatoriums en France (1900-1945)*, de Philippe Grandvoinnet (2014), procurou registrar a construção de sanatórios de tuberculose por toda a França na primeira metade do século XX, indicando a importância do tema em estudos recentes sobre história das ciências e da saúde.

A começar pelo título, *Arquitectura branca*, o livro de Avelãs Nunes (2022) nos faz refletir sobre diversos aspectos da doença, incluindo a origem da relação entre a doença, a arquitetura e a cor. Uma das primeiras vezes que vimos menção a isso no Brasil foi em uma dissertação de mestrado defendida em 2000, com título semelhante, *Peste branca, arquitetura branca*, de Tania Mara Mota Bittencourt, sob orientação de Hugo Segawa, estudo considerado pioneiro. Com certeza há de ter outras publicações pelo mundo que utilizam a expressão “arquitetura branca” para se referir aos espaços construídos para tratar a tuberculose. Mas como se deu essa associação e por que é importante chamar nossa atenção para isso?

Avelãs Nunes (e também Bittencourt, 2000) deixa pistas ao longo de sua obra, conduzindo o leitor a ler todas as páginas e tirar ele mesmo sua conclusão. A primeira referência é com a própria doença, identificada já no século XVIII como peste branca, possivelmente em contraposição à peste negra, flagelo da Idade Média (Silva, Montañes, Claver, 2003) ou como produto da sociedade industrial e urbanizada de fim do século XIX e início do século XX (Dubos, 1952). Outras associações entre a doença e o branco nos levam ao higienismo, doutrina médica em voga na época; ao romantismo com que a doença foi tratada; e, menos óbvia, à própria modernidade arquitetônica que se desejava na época. Avelãs Nunes disseca todas essas associações, mas se detém nessa última, ao trazer à luz os sanatórios construídos em Portugal no largo período entre 1850 e 1970.

O autor justifica seu estudo com a lacuna encontrada em Portugal ao se deter sobre tais edifícios, seja no método, na abrangência, na organização deles, mas, sobretudo, na relação da arquitetura com a medicina, quer no aspecto construtivo, quer no tipológico e social. Há um esforço, mais que compensador, empreendido pelo autor para estudar a doença em seu país sob todos os aspectos, as regiões e os atores envolvidos, trazendo um estudo estimulante e exemplar. Avelãs Nunes percorre desde as primeiras iniciativas empreendidas na Ilha da Madeira, localidade insular famosa por seu ambiente salubre e dedicado à saúde, passando pela região da Guarda, onde surgem os primeiros sanatórios e médicos da era moderna portuguesa, até os anos de renovação das estruturas públicas e dos hospitais portugueses, bem como a preocupação com a conservação deles como memória e patrimônio cultural. Avelãs Nunes também traça, com maestria, a história da doença pelo lado de sua estruturação administrativa, ao historicizar a atuação da Assistência Nacional aos Tuberculosos desde sua origem monárquica até os anos 1970, pós-Estado Novo português.

Nos anos 1930, tentou-se colocar em prática um ambicioso plano de construção de sanatórios por todas as capitais dos distritos portugueses. A construção de apenas alguns desses sanatórios (na própria Ilha da Madeira, em Viseu e no Porto) foi suficiente para trazer ares renovadores à arquitetura portuguesa, tão presa a sua tradição construtiva. Plano semelhante teve o Brasil com Getúlio Vargas (1937-1945), originando sanatórios por quase todos os estados (Costa, 2019). Da mesma forma, a modernidade esteve expressa em cada um dos projetos, a ponto de não se saber mais a partida e a chegada da influência: da doença para a arquitetura (limpa, incolor, fria e sem ornamentos) ou da arquitetura para o tratamento da doença (galerias de cura, escalonadas ou retilíneas; ventilação cruzada; insolação, para tratamentos à base da helioterapia; localização no mar ou na montanha).

A persistente investigação do autor nos conduz aos desafios enfrentados também no Brasil de nos defrontar com a ausência de acervos sistematizados, o que exige determinação tenaz para dar fio condutor a uma “manta de retalhos”. O livro foi dividido em seis capítulos, cada um designado com expressões que nos instigam pela curiosa associação entre a doença e a arquitetura, associando história da medicina e história da arquitetura em um diálogo apurado.

No intuito de desvendar a matriz tipológica dos sanatórios portugueses, Avelãs Nunes nos enriquece com um conjunto de plantas, fachadas e fotografias de interiores em que seus protagonistas, profissionais de saúde e pacientes, humanizam a história do combate a uma doença estigmatizada. Com exemplar desenvoltura, o autor nos aproxima dos grandes debates científicos e políticos coetâneos, ao mesmo tempo que esmiúça os detalhes arquitetônicos e os tipos de materiais adotados nos revestimentos dos sanatórios, destacando as mudanças de padrões ao longo do arco temporal de 120 anos.

As considerações finais revelam a preocupação do autor com a decadência desses edifícios, fadados ao abandono, como que tomados pelo mesmo destino dos doentes que abrigaram, embora demonstre iniciativas de conversão de alguns exemplares para tratamento de doenças outras ou em equipamento hoteleiro. Assim, a leitura do livro conduz à reflexão acerca da preservação dos espaços de cura no mundo lusófono, diante dos desafios pandêmicos e do valor contemporâneo identificado em diversas soluções adotadas nesses edifícios históricos.
